# Acquisition of musical skills and abilities in older adults—results of 12 months of music training

**DOI:** 10.1186/s12877-024-05600-2

**Published:** 2024-12-19

**Authors:** Hannah Losch, Eckart Altenmüller, Damien Marie, Edoardo Passarotto, Clara R. Kretschmer, Daniel S. Scholz, Matthias Kliegel, Tillmann H. C. Krüger, Christopher Sinke, Kristin Jünemann, Clara E. James, Florian Worschech

**Affiliations:** 1 Institute of Music Physiology and Musicians’ Medicine, Hannover University of Music, Drama and Media, Hannover, Germany; 2 Institute for Music Education Research, Hannover University of Music, Drama and Media, Hannover, Germany; 3 Center for Systems Neuroscience, Hannover, Germany; 4 Geneva Musical Minds Lab, Geneva School of Health Sciences, University of Applied Sciences and Arts Western Switzerland HES-SO, Geneva, Switzerland; 5 CIBM Center for Biomedical Imaging, Cognitive and Affective Neuroimaging Section, University of Geneva, Geneva, Switzerland; 6 Division of Clinical Psychology and Sexual Medicine, Department of Psychiatry, Social Psychiatry and Psychotherapy, Hannover Medical School, Hannover, Germany; 7 Department of Musicians’ Health, University of Music Lübeck, Lübeck, Germany; 8 Institute of Medical Psychology, University of Lübeck, Lübeck, Germany; 9 Center for the Interdisciplinary Study of Gerontology and Vulnerability, University of Geneva, Geneva, Switzerland; 10 Department of Neurosciences, University of Padova, Padua, Italy; 11 Department of Experimental Psychology, University of Oxford, Oxford, England; 12 Faculty of Psychology and Educational Sciences, University of Geneva, Geneva, Switzerland

**Keywords:** Musicality, Older adults, Randomized-controlled trial, Skill learning, Music training

## Abstract

**Background:**

Older adults can acquire new skills across different domains. Practicing a musical instrument has been identified as a promising activity for improving cognition, promoting well-being, and inducing brain plasticity in older individuals. However, the mechanisms of these changes are still poorly understood. This study aims to assess musical skill acquisition in musically naïve older adults over one year of practice, focusing on individual factors influencing this process and the relations between musical skills.

**Methods:**

One hundred fifty-six healthy older adults (age = 69.5 years ± 3.2) from Hannover and Geneva with no prior musical training participated in weekly piano practice (PP) or ‘music culture’ (MC) sessions over a one-year period. Baseline assessments included the Cognitive Reserve Index questionnaire (CRIq) and Cognitive Telephone Screening Instrument (CogTel). Musical abilities were measured using piano performance ratings (PP group), music quizzes (MC group), and aptitude tests such as the Beat Alignment Test (BAT), Melodic Discrimination Test (MDT) and Midi Scale Analysis (MSA) at baseline and six-, twelve and 18-month timepoints. The interrelationship between musical abilities was investigated through correlational analyses, and changes impacted through individual characteristics were modeled using Bayesian statistics.

**Results:**

The PP group demonstrated moderate improvements in piano articulation and dynamics, while the MC group achieved higher scores in the music quiz. Modest improvements in MDT and MSA were observed in both groups, with the PP group showing greater progress is MSA. Higher global cognitive functioning and musical sophistication was associated with greater performance in MDT for both groups. We did not identify any links between individual characteristics, like age, CogTel, CRIq, and musical sophistication, and improvement in musical aptitude tests. Changes in different musical aptitude test scores were not correlated, and neither the development of piano skills nor the music quiz correlated with initial performances on the musical aptitude tests.

**Conclusion:**

Musically naïve older adults can acquire diverse musical abilities, which progress independently, suggesting a broad spectrum of musical abilities rather than a single general musical aptitude. Future research should also explore genetic and psychosocial factors influencing musical development.

**Trial Registration:**

The Ethikkomission of the Leibniz Universität Hannover approved the protocol on 14.08.17 (no. 3604–2017), the neuroimaging part and blood sampling was approved by the Hannover Medical School on 07.03.18. The full protocol was approved by the Commission cantonale d’éthique de la recherche de Genève (no. 2016–02224) on 27.02.18 and registered at clinicaltrials.gov on 17.09.18 (NCT03674931, no. 81185).

**Supplementary Information:**

The online version contains supplementary material available at 10.1186/s12877-024-05600-2.

## Introduction

The profound impact of music on various aspects of human cognition, emotion, and well-being has long been recognized [1–3]. Recent research has focused on the benefits of music engagement for aging populations [1, 4, 5]; however, substantial gaps in our understanding of the impact and mechanisms of musical learning in older adults remain.

### Benefits of acquiring skills and abilities in older age

Aging is linked to deteriorations in cognitive functioning that impact learning abilities. These changes include a decline in processing speed, working memory capacity, inhibition and cognitive flexibility [6, 7], as well as increases in the risk of developing age-related diseases (e.g. dementia) [8]. Nevertheless, research has demonstrated that the adult brain retains a considerable degree of neuroplasticity even in advanced age [9], enabling the aging brain to adapt and modify itself in response to new learning experiences. Cognitive functions also remain adaptable, enabling individuals to learn and perform new tasks requiring cognitive effort [10]. The acquisition of new skills and knowledge in later life facilitates the preservation and further development of executive functioning and other cognitive abilities [10–12]; executive function preservation specifically enables older adults to approach novel challenges with a flexible mindset and develop effective strategies for navigating various situations [10].

The 18-month study of Woodard demonstrated in 78 older adults that actively training perceptual-motor control through physical activity could reduce the risk of cognitive decline [12]. A further study of 80 older adults with no history of cognitive impairment from Zinke et al., demonstrated significant improvements in tasks targeting visuospatial, verbal, and executive control aspects of working memory following training over a nine-month period [13]. These improvements in the Zinke study were not only observed in the specific trained tasks but also in other tasks with similar working memory demands. In a third study, transitory increases in gray matter in the hippocampus were noted throughout a three-month juggling training period, despite the observation that older participants demonstrated a lesser degree of proficiency in learning to juggle relative to younger counterparts [14]. In summary, while younger individuals may demonstrate superior learning rates, older adults still have the capacity to acquire cognito-motor abilities and motor skills [15], which, in turn, enhance cognitive and brain functions.

Given that musical activities engage a range of cognitive domains, including executive functions [16], memory [17], perceptual-motor skills [18], and social cognition [1], musical training is a potentially beneficial leisure activity for maintaining cognitive and brain function in older adults. In addition to the cognitive advantages, musical activities have been demonstrated to enhance well-being [19] and physical health [20] in older adults, including those with neurodegenerative conditions. Further, participation in musical activities has been linked to a reduced risk of dementia and cognitive impairment in older adults [21, 22]. This indicates that musical engagement may serve as a multimodal enrichment strategy to maintain cognition and brain health in later life [5].

Cross-sectional studies have demonstrated that older adults who played an instrument throughout their lives exhibited superior performance compared to their less-engaged counterparts across various cognitive domains, including global cognition, working memory, executive functions, language, and visuospatial abilities [23, 24]. For those with no prior musical experience, musical training in later life has been shown to have some similar benefits. Learning to read and play musical notation over a three-month period induced positive effects on visuospatial abilities and neural activation in the fusiform gyrus and superior parietal regions in individuals with no prior musical experience [25]. A study involving 15 weeks of drumming and singing demonstrated enhanced verbal and visual memory functions in eight older women [26]. Four months of piano practice was shown to enhance cognitive functions related to attention and executive functions and some domains of quality of life in 29 healthy older adults [4].

Despite the apparent efficacy of musical training in mitigating age-related cognitive decline and promoting healthy aging, the underlying mechanisms remain poorly understood [27]. The sample sizes of prior studies were often small, and the duration of the interventions was frequently insufficient to allow the interventions to reach their full theoretical potential. It is possible that large inter-individual variation may have exerted a greater influence on the results than the intervention itself. Further, the specific components of musical engagement, such as listening or instrumental practice, may lead to differing cognitive or functional outcomes. In order to gain a full understanding of the mechanisms by which musical engagement promotes healthy aging, it is essential to evaluate the development of musical abilities in healthy older adults over an extended training period.

### The operationalization of musical skills and abilities

Although musical skills and abilities refer to two distinct concepts, the terms are often used interchangeably. A skill is defined as an organized and coordinated sequence of movements that are directed towards a specific outcome. In contrast, abilities are defined as general individual traits or capacities that influence both the acquisition and the performance of a skill [28]. For example, performing a piece on the piano can be seen as a skill acquired over months and years. This is supported by a range of abilities including rhythm and melodic memory, but also non-musical abilities such as processing speed and working memory [29].

The acquisition of a musical skill is a complex undertaking that involves various abilities and follows an as-yet-unknown structure. Seashore [30] posited that the subunits of musical perception can be used to formulate to a multifactorial model influencing discrete musical abilities. In contrast, Wing [31] proposed that musical factors contribute to a general musical intelligence, analogous to Spearman’s concept of general intelligence [32]. There is a long tradition of investigating musical skill and abilities dating back to Michaelis in 1805 [33], resulting in numerous approaches to testing musical skill; these include those developed by Carl Stumpf [34], Seashore [30], Wing [31], and Gordon [35]. Initially, tests focused on aural perception. However, in the 1990s, tests expanded to include music production aspects, such as sight-reading, performances of rehearsed music, playing from memory, and improvisation. This expansion reflected an acknowledgement of the complexity of musical skill and abilities [36]. Modern examinations of musical abilities seek to encompass the entirety of musical production and perception, considering elements such as timbre, pitch, rhythm, dynamics, articulation, and expression [37]. For example, in the ongoing LongGold longitudinal study (accessible at https://longgold.org/), musical skill and abilities are evaluated by tests involving musical listening and discrimination, such as musical emotion discrimination, perception of mistuning, and self-reported musical questionnaire assessments [38].

Examining the development of musical skill and abilities over time is crucial to determine whether musical abilities develop simultaneously as one general musical skill or rather independently as autonomous musical abilities. An understanding of the interconnectivity of musical abilities could have implications for the development of structured and beneficial music lessons for older adults, with the aim to efficiently promote healthy aging.

### Predicting (Musical) skill acquisition

Also, with implications for efficient healthy aging, accurate predictions of the acquisition of musical skill and abilities remains challenging, as numerous factors appear to influence this process. While cognitive and neuronal plasticity are possible in old age, individual factors such as genetic predisposition, age, baseline performance, general intelligence, education, motivation and intensity of training can influence the extent to which brain plasticity, and therefore skill acquisition, is induced [10, 13, 39–41]. Motivation, genetics and general intelligence have also been shown to be specifically relevant to musical skill development [42–48].

### Aim of the study

This study aimed to assess the acquisition of musical skills in musically naïve older adults over one year of practice. To this end, we investigated the changes in musical skill and abilities over one year in two groups: one engaged in active piano practice, while the other participated in music sensitization activities, which included analytical listening as well as theoretical information of music (e.g. styles, structure). Further, our investigation also aimed to determine whether musical abilities develop concurrently, indicating a *general* musical skill, or independently, suggesting separate musical abilities. We also aimed to identify possible associations between individual characteristics, musical sophistication, and the acquisition of musical skill and abilities.

## Methods

### Participants

This study is a secondary analysis of a comprehensive research project (“Train the brain with music”, TBM [49]), for which the primary objective was to evaluate the impact of musical training on cognition, well-being, motor abilities, and neurophysiological parameters in elderly people. For further details, please refer to the published protocol [49]. In this study, 156 healthy retirees (aged 64–76 years) from Hannover and Geneva who participated in the TBM study were analyzed. All participants were native or fluent speakers of either German (Hannover) or French (Geneva). The individuals were right-handed (assessed using the Oldfield procedure [50]), had engaged in no more than six months of musical practice throughout their lives, and were not reliant on hearing aids.

The global cognitive functioning of the participants was screened using the face-to-face version of the Cognitive Telephone Screening Instrument (CogTel [51, 52]); participants who scored below the threshold score of 10 defined a priori (pathological decline) were excluded from further participation. The CogTel consists of six subtests that evaluate prospective memory, verbal short-term memory, working memory, verbal fluency, inductive reasoning, and verbal long-term memory. The scores range from 0 (lowest) to 60 (highest). Additionally, participants were excluded if they had a current or past neurological disease, severe obesity (BMI > 30), cancer, or clinical depression. Individuals who developed signs of mild cognitive impairment during the intervention period (e.g. assessed by strong inter- and intraindividual deviations in cognitive test scores) were excluded from the subsequent analysis. Prior to enrollment, all participants were informed that the objective of the study was to compare two distinct music interventions, both of which had the potential to positively impact cognitive functioning and brain plasticity (single-blind procedure). Participants were only eligible to participate in the study if they agreed in advance to be randomly assigned to one of the two groups, regardless of their preference. The demographic characteristics of the sample are presented in Table 1.

**Table 1:** Demographic information of the sample, matched for age, sex, education and CogTel. CRIq is the cognitive reserve measured by the Cognitive Reserve Index questionnaire (see below)

		Music Culture	Piano Practice
n		82	74
Site (n)	Geneva	32	32
	Hannover	50	42
Age (mean ± SD)		69.8 ± 3.8	69.5 ± 3.1
Sex (n)	Male	31	32
	Female	51	42
Education (%)	Elementary School	0 (0)	2 (1.3)
	Middle School	20 (12.8)	14 (9)
	High School	14 (9)	11 (7.1)
	Bachelor	12 (7.7)	12 (7.7)
	Master	29 (18.6)	29 (18.9)
	PhD	7 (4.5)	6 (3.8)
CogTel (mean ± SD)		32.3 ± 7.3	30.9 ± 7.1
CRIq (mean ± SD)		138.0 ± 17.3	136.9 + 14.8

### Intervention

The participants were stratified by age, sex, education level, and CogTel scores and then randomly assigned to either the piano practice (PP) or the musical culture (MC) groups.

The PP group engaged in one year of weekly piano practice in dyads (two students and one teacher). The MC group participated in weekly seminars in groups of three to six discussing and listening to various music genres and learning about musical styles, acoustics, musical instruments, and the basics of music theory, all without actively creating music. Participants in both groups were encouraged to attend to at least 40 sessions within 12 months and complete approximately 30 min of daily homework five days per week. Participants in the PP group received electronic pianos (Yamaha P-45) for home practice; those in the MC group received reading materials and internet links for listening to music.

The piano courses began with imitation and listening exercises, which were designed to be engaging and assist participants in becoming acquainted with the keyboard while maintaining a relaxed body posture. Furthermore, clapping, singing, and moving to the beat were also integral parts of lessons. These activities embodied a “bodily-holistic” approach shown to enhance the learning process [53], engaging multiple senses and promoting a physical connection to the music. The reading of music notation was progressively introduced, using a methodology specifically developed for older adults based on Jens Schlichting’s “Piano Prima Vista” (Internote GmbH Musikverlag 2013), and the Hall Leonard “Methode de Piano pour Adultes Volume 1 + 2” (ISBN 9789043134378 & 9,789,043,152,037). These primary sources were supplemented with material from other textbooks not specifically addressing older adults, including “A Dozen a Day, Volume 1” (ISBN 9780711954311), “Bastien Piano Basics – Piano Level 1” (ISBN 0849752663), and Manfred Schmitz’s “Jugend-Album für Klavier” (ISBN 9783932587412). Further, music teachers provided transcriptions of preferred musical pieces selected by participants. The dyadic approach was employed to cultivate a supportive learning environment and enhance group interaction, which is shown to be an effective strategy for learning in older adults [54].

The MC group followed a set curriculum, but which included some flexibility to incorporate specific preferences, experiences, and interests of the participants. Only the first three sessions were standardized in the PP group, allowing for individualization in subsequent sessions due to anticipated variability in musical abilities and learning progress. However, several core principles were emphasized to guarantee the consistency of piano instruction: implementation of the provided materials; incorporation of physical warm-up routines; emphasis on attentive listening; practicing bimanual coordination; and music reading (see Supplementary material 1).

Weekly PP and MC sessions lasted 60 min and were conducted by teachers (*N* = 26; 19 PP and 7 MC) who held at least a bachelor’s degree in musical performance and education with piano or a different principal instrument (*N* = 21), music education (*N* = 3), or music theory (*N* = 2). The teachers also had several years of teaching experience in local music universities, received specialized training in teaching older adults, and were supervised by university-level professors of music education and piano pedagogy. The involvement of numerous teachers helped avoid the influence of a specific teaching style. In the second half of the study, the global COVID-19 pandemic led to a temporary conversion to online intervention delivery. However, for all participants, at least the initial two measurements (T0 and T1, described below) were unaffected by the pandemic.

### Measurements

**Fig. 1:**
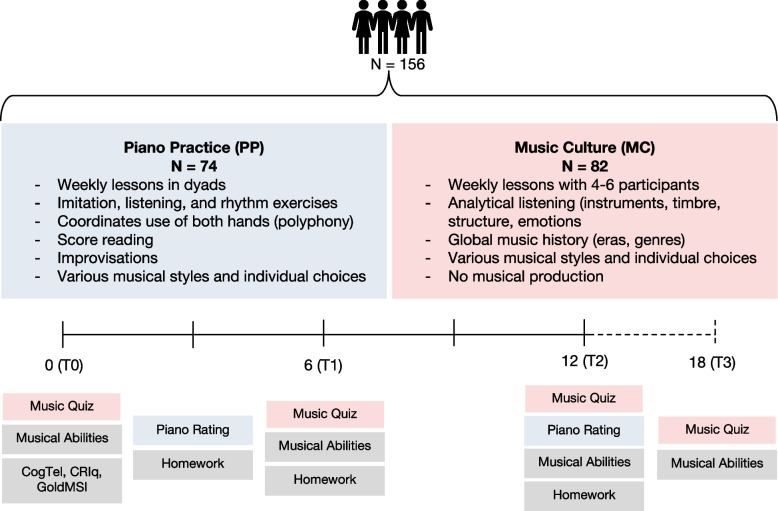
Intervention process and testing schedule of 18 months throughout the intervention. Musical Abilities includes BAT, MDT, MSA, and MSA_pp_

Outcome data were acquired at baseline (T0) and at 6 months (T1), 12 months (T2), and six months follow-up (T3) timepoints. Three months into the intervention, participants were asked to report the amount of time they spend on homework and practice at home. This information was also collected at T1 and T2. To assess the progress of piano performance in the PP group, recordings of a specific musical piece were made after three and twelve months of practice. Figure 1 shows the intervention process and testing schedule.

### CRIq (Cognitive Reserve Index questionnaire)

The Cognitive Reserve Index questionnaire (CRIq) was used to assess the cognitive reserve of the participants. This was based on their educational background, work experience, and frequency of engaging in leisure activities, such as sports, culture, and travel. A score below 70 indicates a very low cognitive reserve, while a score above 130 indicates a high cognitive reserve [55].

### Gold-MSI (Goldsmiths Musical Sophistication Index)

Participants completed the Goldsmiths Musical Sophistication Index (Gold-MSI [56]) to assess their ability to engage with music. This self-report inventory assesses various facets of musical sophistication, including active engagement, perceptual abilities, musical training, self-estimated singing abilities, emotional response to music, and a general musical sophistication score. The Gold-MSI scores are presented in Table 2.

**Table 2:** Gold-MSI scores of the sample

	Piano Practice	Music Culture	Group difference, t-test (t, df, p)	Norms^a^
Active Engagement	30.3 (8.7)	29.9 (8.1)	−1.1, 142.4, 0.3	41.5 (10.4)
Perceptual Abilities	40.1 (9.9)	40.2 (8.3)	0.4,147.4, 0.7	50.2 (7.9)
Musical Training	12.3 (4.6)	11.3 (3.6)	0.9, 150, 0.4	26.5 (11.4)
Singing Abilities	23.4 (6.9)	21.9 (6.3)	−0.5, 146.8, 0.7	31.7 (8.7)
Emotions	27.8 (6.6)	28.0 (5.6)	0.1, 146.2, 0.9	34.7 (5.0)
General Musical Sophistication	53.8 (14.4)	52.5 (11.9)	0.3, 147.7, 0.8	81.6 (20.6)

^a^Norms from Müllsensiefen et al. [56], *N* = 147,633 participants from the UK aged 35.2 (SD = 15) years

### BAT (Beat Alignment Test)

The BAT assessed participants’ ability to perceive a beat. In a two-alternative forced-choice task, participants were required to determine which of two presented beeps is synchronized with the musical stimulus. The test comprised 25 trials and was conducted using *Sennheiser HD380pro* headphones. The resulting value is the standard error of measurement for the participant’s ability estimate, calculated from the underlying item response model [57].

### MDT (Melodic Discrimination Test)

The MDT is a tool used to assess an individual’s capacity to discern subtle melodic variations. The participants were asked to identify single note differences among three transposed versions of the same melody. The 20 stimuli were presented using *Sennheiser HD380pro* headphones. The standard error of ability measurement is computed from the underlying item response model [58].

### MSA (MIDI Scale Analysis)

The MSA measured the ability to play a five-note scale on the piano. The task was initially introduced by Jabusch et al. [59] as a diagnostic tool for musician’s dystonia, a neurological movement disorder. This measure has been demonstrated to be a reliable indicator of basic piano performance [60]. The MSA entails the performance of the initial five notes of the C major scale (C-D-E–F-G-F-E-D-C) with each finger of the right hand at a tempo of 76 beats per minute, with one note played per beat (MSA). The PP group additionally performed the five-note scale at double tempo, with two notes played per beat (MSA_pp_). The inter-onset interval was calculated as the average deviation from the time between the onsets of two subsequent notes. A MSA score of 0 would indicate that the participant’s performance was perfectly smooth in terms of temporal aspects.

### Group-specific outcomes

#### MQ_MC_ (Music Quiz)

The learning progress of the MC group was measured through in-house music quizzes. These quizzes consisted of 15 questions, of which five pertained to musical knowledge, including definitions of musical terms, and 10 were listening tasks. For examples, please refer to Supplementary material 2.

#### Piano Recordings

The progress of piano learning within the PP group was evaluated through MIDI recordings at three- and twelve-month (T2) timepoints. At both timepoints, participants performed a simplified version of Beethoven’s "Ode to Joy" (see Supplementary material 3). The three-month recording was the earliest time-point at which bimanual coordination was trained to a sufficient extent, with the left hand able to perform sustained notes on “C” and “G”. In addition to the easy version, the participants performed a more difficult version of the “Ode to Joy” at the twelve-month mark. The more advanced piece featured a more complex yet still simplified left-hand voicing (see Supplementary material 3). The more complex version was introduced to avoid anticipated ceiling effects and provide a challenge for the participants. During the recording, the participants were encouraged to play continuously without restarting and follow the instructions provided on the sheet music, including dynamics and articulation.

#### Rating procedure for piano recordings

The piano recordings were evaluated systematically by nine raters. The raters were between the ages of 20 and 30 (M = 25.78, SD = 3.23) and had considerable experience with piano practice (M = 18.1 years, SD = 2.71 years). Six of the raters held at least a bachelor’s degree in piano, while two had a degree in music education and one a master’s degree in psychology. The raters were instructed to evaluate the 3- and 12-months recordings randomly ordered solely based on a set of predefined musical parameters, including articulation, rhythm, dynamics, pitch, fluency, and expressivity. For a detailed overview of these parameters, refer to Supplementary material 4. Ratings were assigned on a scale ranging of 1 to 7. The raters listened to each recording twice in the context of two ‘runs’. During the initial run, raters evaluated the recordings based on three criteria: articulation, rhythm, and dynamics. During the second run, they focused on pitch, fluency, and expressivity. At the end of each run, the raters rated 30 recordings again to ensure the reliability of the ratings. Each of the two runs consisted of five sessions, with each session comprising approximately 25 excerpts of the “Ode to Joy” (total time: ∼ 25 min). The raters performed a maximum of two sessions per day. The two runs were completed within two to four weeks.

Interrater correlation of piano performance ratings was calculated using a two-way mixed effects model, as defined by Shrout and Fleiss [61]. Intraclass correlation coefficients based on double-rated recordings were computed to evaluate the reliability of each rater (see Supplementary material 5). Both coefficients were derived by subtracting the variance between subjects from the residual variance and dividing the result by the variance between subjects [61].

### Statistics

All statistical analyses were conducted in R [62] using the ‘brms’ package [63, 64]. Bayesian inference was selected as the framework for quantifying uncertainty in parameter estimation, with all reported effects accompanied by 95% credible intervals (CI). All models allowed the slopes and intercepts to vary across participants. The intercept represents the baseline performance at the beginning of the intervention (T0), while the slopes represent the change in performance over the course of the study. Information regarding model convergence was obtained from Rhat values (a function that compares the between- and within-chain estimates for model parameters [65]), with Rhat < 1.1 indicating satisfactory convergence. To ensure an optimal fit, trace plots (time series plots used to visualize the mixing of chains throughout the sampling) were examined, and posterior predictive checks were conducted using the pp_check R-function. Prior to analysis, all variables and demographic predictors were centered at their respective means and scaled. Accordingly, a one-unit change is equivalent to a change of one standard deviation. Dummy variables (0|1) were used to encode sex (female|male) and site (Hannover|Geneva).

A Bayesian multilevel model with a beta distribution was employed to analyze piano ratings of the simple version of “Ode to Joy”. Given that the beta distribution is defined within the interval (0,1), 1 to 7 ratings across all 6 parameters were scaled down by a factor of seven. This ensured that the highest conceivable rating corresponded to 1 and the lowest to 0. A Bayesian multilevel approach was employed for each musical parameter (articulation, dynamics, rhythm, fluency, pitch, expressivity) as a dependent variable using the regression equation below. The intercepts and slopes were allowed to vary across both participants and raters. Additionally, each musical parameter was weighted by the rater’s intra-class correlation coefficient (ICC), so ratings from reliable raters (high ICC) were given more weight than those from less reliable raters:

$$Variable|weight\left(rate{r}_{icc}\right)\sim time+\left(1+time|ID\right)+(1+time|rater)$$

Similarly, MQ_MC_, BAT and MDT were analyzed using Gaussian mixed effects models, accounting for group differences (MC and PP). To assess changes over the course of each six-month period, we used linear splines:

$${MQ}_{MC}\ or\ BAT \ \text{or }MDT\sim\ time*group+(1+time|ID)$$

The Midi Scale Analysis scores were analyzed using an exponential model. The baseline score ($\alpha$), asymptote ($\beta$), and learning rate ($\gamma$) were allowed to correlate and to vary across participants:

$$MSA \ \text{or }{MSA}_{pp}\sim \beta +\left(\alpha -\beta \right)*\mathit{exp}\left(-\mathit{exp}\left(\gamma \right)*time\right),$$

*with*

$$\alpha \sim 1+group+\left(1\left|time\right|ID\right)$$

$$\beta \sim 1+group+\left(1\left|time\right|ID\right)$$

$$\gamma \sim 1+group+\left(1\left|time\right|ID\right)$$

In the second step, all outcome variables (intercept and slope) were correlated to investigate the interconnectivity of the various musical abilities that were assessed. In cases where variables exhibited high correlation, exploratory factor analysis was employed.

To predict the acquisition of musical skill and abilities, interaction effects between individual characteristics (age, sex, CogTel, musical sophistication, and cognitive reserve) and time were incorporated into the models, accounting for baseline correction and changes over time. Resulting from the factor analysis, the variables for the piano ratings were here unified as one *piano progress* factor. The models were modified as follows:

$$Piano\ Progress\sim individual\ characteristic*time+\left(1+time|ID\right)+(1+time|rater)$$

$${MQ}_{MC} or BAT \text{or }MDT\sim time*group+individual\, characteristic+(1+time|ID)$$

MSA and MSA_pp_ were analyzed independently with respect to the individual characteristics mentioned above.

## Results

Eleven participants (3 PP, 8 MC) left the study between T0 and T1, ten participants (1 PP, 9 MC) left between T1 and T2, and 35 participants (16 PP, 19 MC) left between T2 and T3. Based on the cognitive assessments, no participant showed cognitive changes beyond age-related decline that could indicate mild cognitive impairment. Teachers did not report any cognitive issues with their students. Furthermore, no abnormal brain atrophy was observed in the MRI data. All statistical models used in the analyses exhibited satisfactory convergence with Rhat-values of 1.0.

### Changes of Musical Skill and Abilities Throughout the Intervention

#### Piano Recordings

**Fig. 2:**
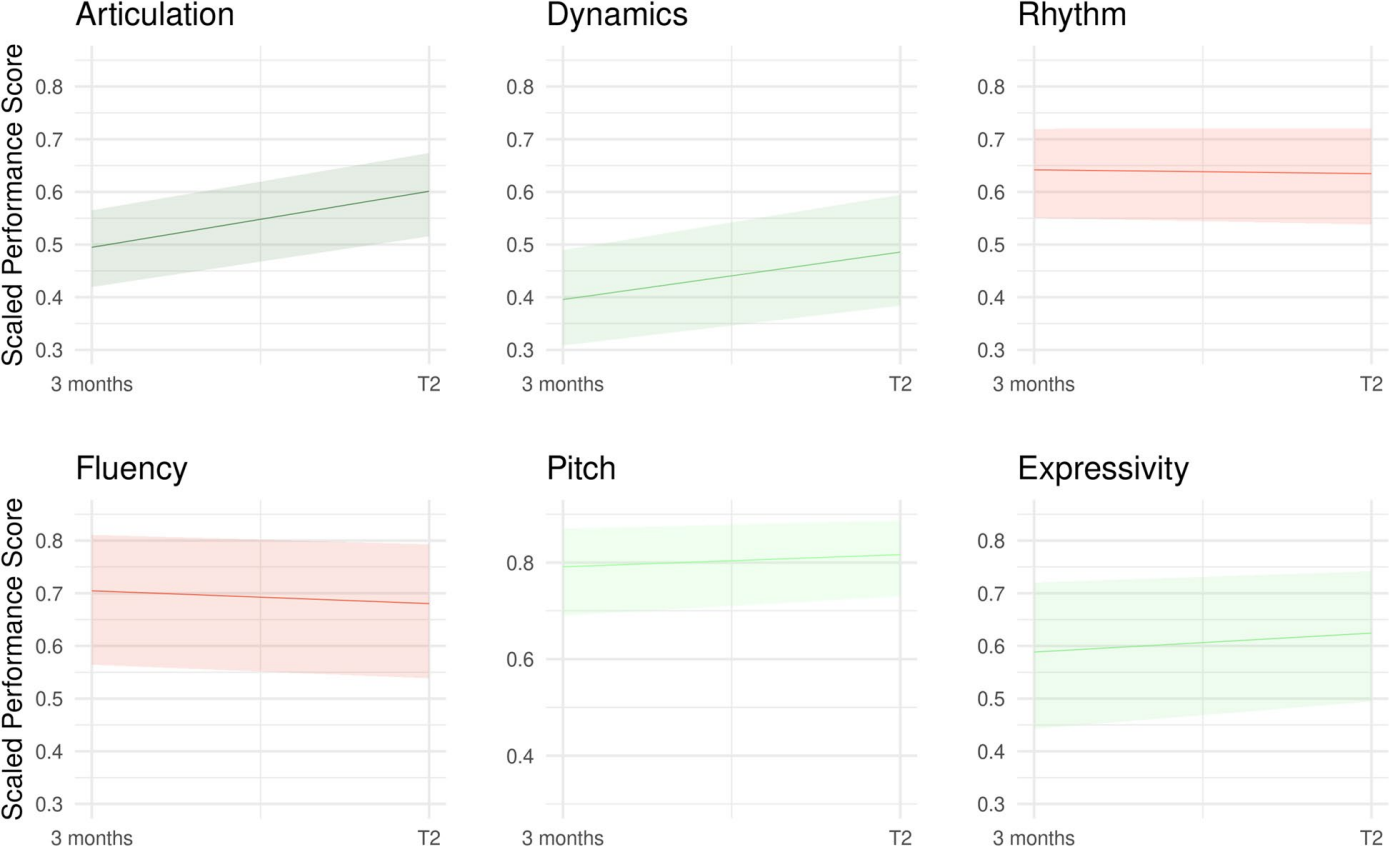
Trajectories of the six musical parameters of piano recordings from three months to 12 months (T2) of piano practice

The analysis of piano recordings across the six musical parameters revealed generally strong variations in slopes and intercepts (see Fig. 2). Within this variation, articulation exhibited the most pronounced improvement (0.06 [0.00, 0.13]) from 3 to 12-month timepoints. Dynamic quality tended to improve 0.05 [−0.01, 0.11], however fluency scores tended to decrease (−0.03, [−0.08, 0.01]), and rhythm (−0.01 [−0.03, 0.05]), pitch accuracy (0.01 [−0.04, 0.05]), and expressivity (0.02 [−0.03, 0.06]) did not change over the course of the study.

#### MQ_MC_

**Fig. 3:**
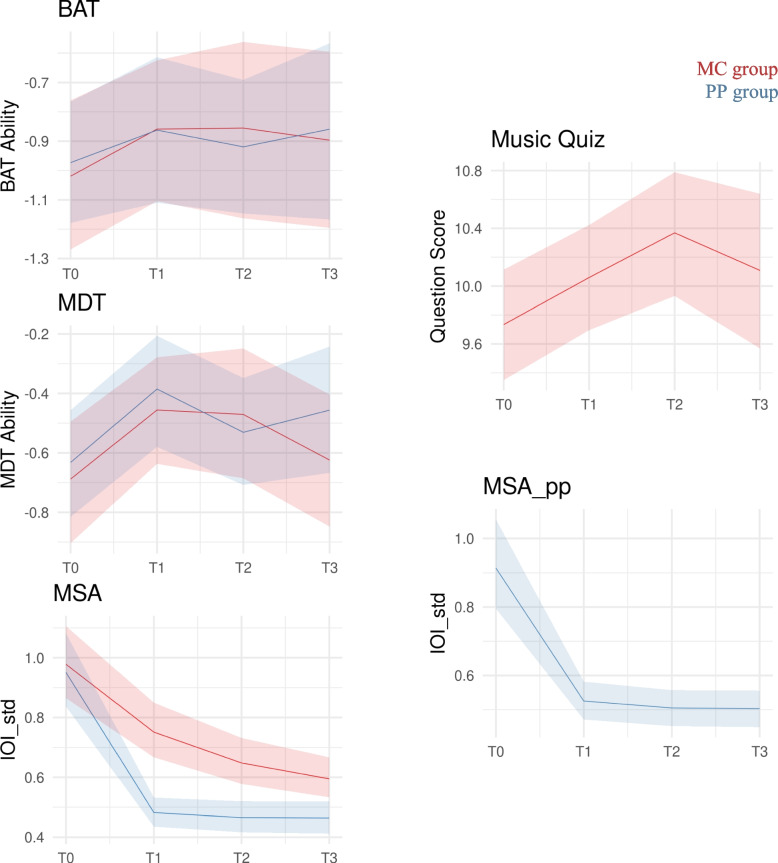
BAT, MDT, MQ_MC_, MSA, and MSA_pp_ changes from the baseline (T0) to the six-month (T1) and 12-month (T2) intervention periods, and 6-month follow-up (T3). The red data points represent the MC group, while the blue data points represent the PP group

The MQ_MC_ scores of the MC group improved over the course of the twelve-month intervention (combined six- and twelve-month time effects: 0.63 [0.2, 1.07]). Once the classes concluded, some of these knowledge gains tended to reverse (T2 to T3: −0.26 [−0.86, 0.35]), although with significant variance across participants (Fig. 3, right top).

#### Beat alignment test

Beat alignment scores did not change throughout the intervention.

#### Melodic discrimination test

Melodic discrimination improved in both groups from T0 to T1 (0.23 [0.02, 0.44]), with a slightly smaller increase in the PP group. While from T1 to T2, MDT scores in both groups remained relatively stable, from T2 to T3, the MC group experienced a decline, while the PP group scores tended to increase in (0.23 [−0.05, 0.51]).

#### Midi scale analysis

Although MC improved, PP showed a greater learning rate (1.83 [0.97, 2.71]). In comparison to MC group, PP group achieved lower asymptotic performance (−0.21 [−0.36, −0.06]), reaching a lower inter-onset deviation between notes.

#### Midi scale analysis piano practice

PP improved also in the double-time version of MSA, with a positive learning rate of (1.00 [0.56, 1.50]).

### Interconnectivity of musical abilities

The baseline scores of the six musical parameters of piano recordings exhibited a robust correlation, suggesting one underlying general *piano playing skill*. Exploratory factor analysis revealed the presence of a single latent factor, hereafter referred to as *piano playing skill* (refer to the Supplementary material 6 for additional details on factor analysis). When removing the more refined skills expressivity and dynamics from the *piano playing skill* model, the fit improved from 0.89 to a very good fit of 0.98 in Comparative Fit Index (CFI) and Tucker-Lewis Index (TLI) and from 0.18 to 0.11 in Root Mean Square Error of Approximation (RSMEA) [66]. Therefore, we decided that the latent factor *piano playing skill* was driven by only four musical parameters: articulation, rhythm, pitch, and fluency.

**Fig. 4:**
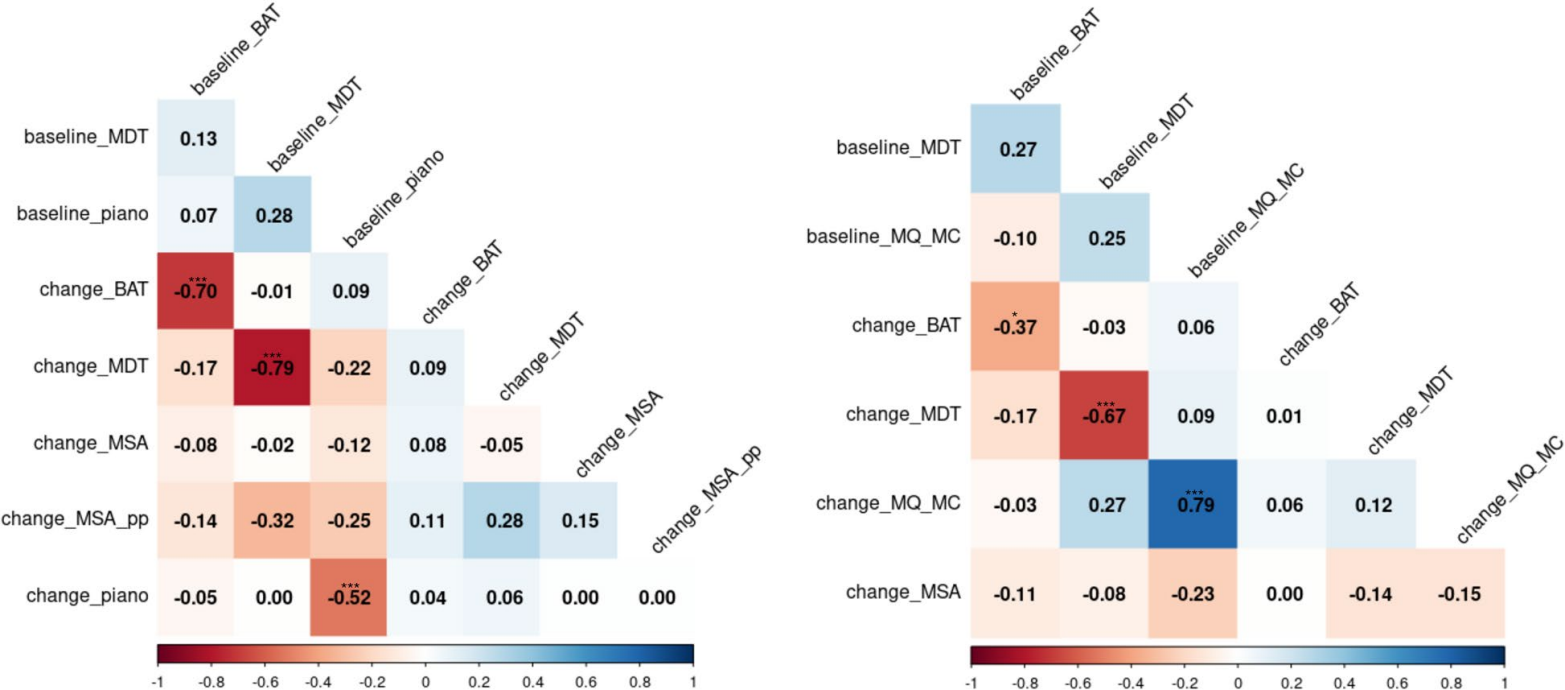
Correlation coefficients of PP (left) and MC (right) group with ***0.001, **0.01, *0.05 significance levels

No meaningful associations were identified between the variables (BAT, MDT, MSA, MSA_pp_, piano playing progress, and MQ_MC_), nor between their change scores through correlational analysis (Fig. 4).

### Associations between individual characteristics and acquisition of musical skills and abilities

For this analysis statistical models presented in the preceding results sections were expanded to include the following variables: age; CogTel scores; sex; cognitive reserve; musical sophistication; and time spent on intervention homework.

*Piano Playing Skill, MQ*_*MC*_ *, MSA, MSA*_*pp*_ *:* No individual characteristics were associated with the progression in any of these domains.

*BAT:* Being male had a positive effect on baseline BAT scores (0.47 [0.16, 0.77]). However, it had no influence on the progress of BAT over time.

*MDT:* Greater CogTel scores and musical sophistication tended to influence MDT baseline scores (CogTel: (0.16 [−0.03, 0.34]; musical sophistication (0.39 [−0.10, 0.87]). Conversely, these predictors did not influence the change of MDT scores over time.

## Discussion

Prior studies indicate that older individuals retain the capacity to acquire new skills [28]. Twelve months of piano training in the present study demonstrated this capacity. However, musical skills and abilities were not universally improved and individual characteristics were not associated with musical progress, both of which have implications for future research and possibilities for broad implementation.

Twelve months of piano training led to clear progress in piano articulation and dynamics, although piano playing fluency decreased over the year-long practice period. The latter finding is counter-intuitive and may be explained by the introduction of a similar but more challenging version of "Ode to Joy" after 12 months of training, which may have resulted in interference between the two versions performed at T1 and T2.

The absence of a positive trend in rhythm development is also somewhat counter-intuitive, as rhythm is typically expected to develop more easily in beginners [67]. The relatively stable BAT scores in both groups throughout the intervention raises questions about the adaptability of older adults to rhythmic tasks. The multifaceted nature of rhythm perception and production may involve genetic and cognitive factors [47, 68] that interact differently with the aging process, emphasizing the complexity of rhythmic skill development.

The initial slightly higher increase in MDT scores within the PP group suggests a beneficial impact of piano training on participants’ capacity to differentiate between melodies. This may be at least partly explained by an improvement in working memory, as shown in recent publications related to the same longitudinal study [69, 70]. After the initial six-month period, however, the MDT scores stabilized and did not improve further, indicating a more complex, non-linear relationship between musical training and melody discrimination and suggesting potential ceiling effects. The comparable improvements in the PP and MC groups suggest that analytic listening to music can yield similar improvements in music perception abilities as playing an instrument.

The substantial time-group interaction effect in MSA scores we found unsurprisingly indicates that piano practice contributes to a more precise temporal execution of piano sequences. This replicates in seniors findings from longitudinal studies of piano practice in younger students [60].

The factor analysis of the six musical parameters in piano recordings revealed the presence of a single overarching piano playing skill. However, a correlational analysis of all musical aptitude tests indicated that musical domains are largely independent, thereby refuting the existence of a generalized musical skill. The structure of human musicality thus appears to diverge from the structure of human cognition as delineated in Carroll’s three-stratum theory [71], and instead seems to align more closely with the Cattell-Horn-Carroll theory of cognitive abilities [72]. In light of this theory, melody discrimination, beat alignment, and piano playing skill could represent broad abilities, which are themselves constituted by narrow abilities. The factor analysis indicated that the narrow abilities strongly associated with piano playing skills are rhythm, fluency, articulation, and pitch accuracy. Expressivity, which exhibited high variance, did not fit into the piano playing skill modeled in the present study, perhaps due to its subjective and complex nature. It is also likely that participants had not yet developed sufficient technical proficiency in their playing to fully express musical nuances. Meanwhile, dynamics may have been perceived more as a sensorimotor task by our participants, differentiating it from the other aspects and resulting in its exclusion from the model. Moreover, our still beginner pianists were likely focused on simply playing the correct notes and were not yet able to manage to additional challenge of expressing dynamics.

The lack of correlation between rhythm perception, melodic discrimination, piano playing, and musical knowledge indicates that these abilities may evolve independently, supporting Seashores’ idea of distinct musical abilities that do not contribute to one general musical intelligence [30]. However, further research needs to be carried out before definitive conclusions about the structure of human musicality can be drawn.

Demographic variables appeared to influence only a select set of musical abilities at baseline; the progression of any musical skill or abilities over the 12-month intervention was not substantively linked to any individual characteristics. Future research is needed.

In conclusion, the study shows that older non-musicians can indeed acquire musical skill and abilities. Moreover, the study suggests that musical abilities develop independently in older adults, which may allow for targeted training of different musical aspects. Future research should focus on further clarifying the independent development of these musical abilities, as well as further endeavoring to identify individual factors which may impact musical development.

### Strengths and Limitations

The study showed that 156 older non-musicians were able to acquire new musical abilities over the course of a year through either piano practice or musical culture lessons. Due to the global pandemic, some of the lessons were conducted online and may have led to a reduction in the intensity of teaching and learning activities and thus the reported effects may be smaller than with fully in-person instruction.

It should be noted that the musical abilities tested in this study represent only a subset of the full range of musical abilities that can be measured. For example, musical emotion discrimination and improvisation ability are not included in the study. Further investigation is needed to ascertain whether these finding can be replicated in younger populations or individuals with different musical backgrounds. Although the duration of the intervention provides a comprehensive view of how musical abilities develop over time in older adults, the impact of the global pandemic and the introduction of a slightly more difficult version of “Ode to Joy” may have interfered with participants’ performance, leading to increased variability in learning outcomes. Further exploration of predictors for musical learning could be conducted by considering additional variables such as motivation, genetic factors, physiological changes and other psychosocial factors. Finally, although one year is a considerable period for an interventional study, it is still far too short to fully develop the participants’ instrumental and perceptual musical abilities, especially in the context of older adults.

## Supplementary Information

Supplementary Material 1.

Supplementary Material 2.

Supplementary Material 3.

Supplementary Material 4.

Supplementary Material 5.

Supplementary Material 6.

## Data Availability

The raw data supporting the conclusions of this article will be openly available at https://osf.io/dzh5b.
