# Supplementary material for: Acquisition of musical skills and abilities in older adults—results of 12 months of music training
Source: BMC Geriatr. 2024 Dec 19;24:1018. doi: 10.1186/s12877-024-05600-2 (PMC11658158; doi:10.1186/s12877-024-05600-2)
Supplement: Supplementary file 1 — Supplementary Material 1. [file 12877_2024_5600_MOESM1_ESM.pdf]

First Six Months Curriculum for Playing Piano

Each participant received an electronic piano, adjustable stool, and headphones for homework[1].

Courses: In the beginning, the participants performed imitation and listening exercises guided by the teacher. Most of them were playful and allowed the participants to familiarize themselves with the keyboard and adopt a correct and relaxed body posture.

The three essential components of the courses are:

1. (10 min) Warming-up, posture exercises, exploring the keyboard
2. (40 min) Learning to play the piano, alone and together, with and without accompaniment, first with one hand (alternating right and left), then with both hands; first only by ear, later on also by reading a score; improvisation; constant reminders of correct body posture
3. (10 min) Precise homework instructions

The pillars for learning to play the piano are: 1) listening, 2) sensory and motor skills, 3) rhythm, 4) producing musical idiom (through listening or through score reading), 5) experiencing pleasure, 6) creativity (within a musical piece or by improvisation)

Month 1

Examples of exercises:

- Dissociate white and black keys; twins and triplets; the seven full octaves; high vs. low; soft vs. loud

[1]Yamaha Germany & Yamaha Switzerland generously offered the electronic pianos (Yamaha P-45 B)

- Hit a key with different strengths; listen carefully, "Genie[2]","Kangaroo[3]", and "Feeling dust[4]" exercises with variations; play motives, for example, the "Telecom-Motive" (CCCEC) or Beethoven 5th (EEEC) in all octaves
- Initiation to improvisation. Example: improvise pentatonic melodies on black keys; dialogical-improvising (question-answer);
- Include the pedal right away
- Singing and playing alternately; translating rhythms into spoken text; playing silently on top of the piano with correct hand position; clapping, singing or walking within a certain rhythm

Homework

Correct posture: sitting; distance to the keyboard; leg position; finger and hand position, Genie, Kangaroo exercises (these exercises should remain in the exercise program for a long time and ultimately be practiced with both hands and all fingers); improvisation on black or white keys; try to play a simple song by ear on the piano that was learned in class but also new ones, even with eyes closed.

Month 2-4

[2]A self-selected key in the lower middle position is initially hit as softly as possible while holding the pedal. This is followed by calm repetitions of notes with a crescendo that is as finely graduated as possible up to a justifiable fortissimo (from “not yet sound” to “no longer sound”) and back again.

[3] Here, all keys with identical note names should be hit in a steady but constant meter, one octave apart, from bottom to top and back. The left-hand plays on the left half of the keyboard, the right hand on the right half. This exercise can be varied and expanded in many ways: with the pedal; with different fingers; with additional tones (intervals smaller than an octave).

[4]With an upright posture while sitting at the piano, use all ten fingertips to feel the imagined dust alternately on the level of the keys and on the level of the music stand. The movement from one level to the other should be elegant and characterized by smoothness in the shoulders, elbows, and wrists. Also, with eyes closed.

New exercises: “dolphin[5]", “mountain[6]"

All exercises (genie, kangaroo, feeling dust, dolphin, mountain) are repeated regularly throughout the first 6 months of training with many variations. By means of that, body posture and freedom of movement are continuously trained. This also holds for motive imitation and playing by imitation (by ear).

Regular playing with eyes closed.

The individual piano teachers introduced music reading progressively using a method specifically developed for our elderly population based on Jens Schlichting’s “Piano Prima Vista” (Internote GmbH Musikverlag 2013), and the Hall Leonard piano method for adult’s volume I (ISBN: 9789043134378). Both methods exist in German and French.

Music reading was introduced later or earlier by individual piano teachers, some only taught by listening and imitation during this period. Other materials:

- Simple pieces of the “Jugendalbum für Klavier” by Manfred Schmitz (ISBN:9789043134378) • “A dozen a Day”, volume 1 (ISBN: 9780711954311);
- A simple arrangement of "Ode to joy" (score provided below)
- Improvisation using different triggers: mood, drawings, a motive.

Homework

All exercises, new and old pieces trained during the courses, improvisation.

Month 5-6

---

[5] Variation of the kangaroo: the selected notes are played legato alternately with both hands – initially again at octave intervals, so that one hand “dives” and the other “flies”. Here, too, the octaves can be filled in later, so that, for example, broken chords are created.

[6] Each finger is placed in its theoretically ideal position on the key (or a solid surface), so that the knuckle forms the highest point with fingers 2 to 5 and the underside of the wrist and fingertips are at approximately the same height. The fingers are stable and round. In this position, the finger is now stressed for several seconds, then: conscious, quick and complete loosening.

All exercises (genie, kangaroo, feeling dust, dolphin, mountain) are repeated regularly throughout the first 6 months of training with many variations. By that means, body posture and freedom of movement are continuously trained. This also holds for motive imitation and playing by imitation (by ear).

Regular playing with eyes closed. Other materials:

- Jens Schlichting's "Piano Prima Vista" (Internote GmbH Musikverlag 2013)
- Transcriptions of favorite pieces of the participants, arranged by the music teachers (Amélie Poulenc (Yann Tiersen); Dvorak Symphony "From the New World", etc.)
- More complicated pieces of the "Jugend-album für Klavier" by Manfred Schmitz (ISBN: 9789043134378)
- Hall Leonard piano method for adults, volume II (ISBN: 9789043152037).

Learning some basics of music theory, tonality, half and whole tones, chord progressions. Playing in front of the other participant on a voluntary basis.
Homework All exercises, new and old pieces trained during the courses, improvisation.
