# Supplementary material for: Acquisition of musical skills and abilities in older adults—results of 12 months of music training
Source: BMC Geriatr. 2024 Dec 19;24:1018. doi: 10.1186/s12877-024-05600-2 (PMC11658158; doi:10.1186/s12877-024-05600-2)
Supplement: Supplementary file 2 — Supplementary Material 2. [file 12877_2024_5600_MOESM2_ESM.pdf]

Music Questionnaire for Music Culture Group

##	Frage	Antwort
	**Wissensfragen**	
1	Zu den Komponisten aus Periode der Wiener Klassik gehört **nicht:**	
	a) Joseph Haydn<br>b) W.A. Mozart<br>c) L. v. Beethoven<br>d) Robert Schumann	
2	Zu den Holzblasinstrumenten gehört **nicht:**	
	a) Querflöte<br>b) Klarinette<br>c) Oboe<br>d) Waldhorn	
3	Musik hören kann **nicht** folgende körperliche oder geistige Effekte haben:	
	a) Verbesserung der Altersweitsichtigkeit<br>b) Beeinflussung des Schwitzens<br>c) Gänsehaut<br>d) Beeinflussung der Herzrate	
4	Welcher der nachfolgenden Komponisten hat die Melodie unserer Nationalhymne komponiert?	
	a) Ludwig van Beethoven<br>b) W.A. Mozart<br>c) Joseph Haydn<br>d) Johannes Brahms	
5	Welcher der nachfolgenden Komponisten hat die Melodie der Europahymnekomponiert?	
	a) Edward Elgar<br>b) Georg Friedrich Händel<br>c) Ludwig van Beethoven<br>d) Franz Schubert	
6	Welches der 4 genannten Musikinstrumente hat **nicht** den Nachnamen seinesErfinders im Instrumentennamen?	
	a) Saxophon<br>b) Sarrusophon<br>c) Sousaphon<br>d) Chalumeau	
7	Was versteht man unter „Minimal Music“?	
	a) Musik, die von einem Instrument gespielt wird<br>b) Musik, die elektronisch erzeugt wird<br>c) Musik, die durch Wiederholung und Überlagerung von Motiven entsteht<br>d) Musik, die besonders leise ist	

8	In welcher der folgenden musikalischen Gattungen kommt **keine** Arie vor?	
	a) Oratorium<br>b) Sinfonie<br>c) Kantate<br>d) Oper	
9	Welchen Schlüssel gibt es **nicht**?	
	a) Celloschlüssel<br>b) Violinschlüssel<br>c) Tenorschlüssel<br>d) Bassschlüssel	
10	Clara Schumann war Robert Schumanns…	
	a) Schwester<br>b) Mutter<br>c) Ehefrau<br>d) Tochter	
11	Was bedeutet Polyphonie?	
	a) Mehrstimmigkeit<br>b) Rhythmische Dichte<br>c) Rhythmische Eintönigkeit<br>d) Einstimmigkeit	
12	Aus welchem Land stammt die Popgruppe ABBA?	
	a) Südafrika<br>b) Schweden<br>c) USA<br>d) Italien	
13	Welches der folgenden Instrumente gehört **nicht** zu den Tasteninstrumenten?	
	a) Cembalo<br>b) Hapsichord<br>c) Xylophon<br>d) Orgel	
14	Welche Komponistin/ welcher Komponist gilt als Hauptvertreter desImpressionismus?	
	a) Lilli Boulanger<br>b) Gabriel Faure<br>c) Claude Debussy<br>d) Eduard Lalo	
15	Welcher der nachfolgenden Musiker gehörte **nicht** zu den „Beatles“?	
	a) Pete Best<br>b) George Harrison<br>c) Ringo Starr<br>d) Eric Clapton	
16	Warum hat Schuberts 8. Symphonie in h-Moll den Beinamen „Die Unvollendete“ bekommen?	

	a) weil F. Schubert während des Komponierens gestorben ist<br>b) weil F. Schubert durch die Arbeit an einem anderen Werk abgelenkt war<br>c) weil diese Sinfonie nur aus 2 Sätzen besteht<br>d) weil diese Symphonie mitten im 2. Satz abbricht, der nicht zu Ende komponiert wurde	
17	Welche der folgenden Stücke ist **keine** Sinfonie von Mozart?	
	a) „Pariser“ Sinfonie<br>b) „Linzer“ Sinfonie<br>c) „Berliner“ Sinfonie<br>d) „Prager“ Sinfonie	
18	Eine Tonleiter mit 5 Tönen bezeichnet man als:	
	a) Quintatonik<br>b) Dur-Tonleiter<br>c) Pentatonik<br>d) Ganztonleiter	
19	Welcher der folgenden Begriffe bezeichnet **keine** Rockmusik-Gattung?	
	a) Grief<br>b) Grunge<br>c) Crossover<br>d) Punk	
20	Bei welchem berühmten Komponisten hatte Ludwig van Beethoven Unterricht?	
	a) Haydn<br>b) Bruckner<br>c) Brahms<br>d) Chopin	
	**Zusatzfragen:**	
E1	Welche der folgenden Bands stammt **nicht** aus der DDR?	
	a) Karat<br>b) Puhdys<br>c) Panikorchester<br>d) City	
E2	Was bezeichnet man in der Musik als „klassische“ Musik?	
	a) Sogenannte „E - Musik“ im Gegensatz zur „U - Musik“<br>b) Musik, die etwa zwischen 1770 und 1820 komponiert wurde<br>c) Musik, die als zeitlos gültig angesehen wird<br>d) einen Kanon von wertvollen Kompositionen aus der Vergangenheit	
E3	Was bedeutet „crescendo poco a poco“ in der Musik?	
	a) allmählich ausdrucksvoller spielen<br>b) allmählich lauter werden<br>c) allmählich schneller werden<br>d) allmählich leiser werden	
	**Höraufgaben:**	

21	Mozart: Kleine Nachtmusik – 2. Satz in der Quartettversion (0:00 – 0:30)<br>https://www.youtube.com/watch?v=XHd6bMka8co	
21	Was für Instrumente spielen mit?	
	a) Streichinstrumente<br>b) Zupfinstrumente<br>c) Holzblasinstrumente<br>d) Blechblasinstrumente	
22	West Coast Hip Hop (Xzibit, etc, 0:00 – 0:30)<br>https://www.youtube.com/watch?v=Vl_uvm6MHnU	
22	Um welche Musikgattung handelt es sich hier:	
	a) Dance<br>b) Soul<br>c) Funk<br>d) Hip Hop	
23	Lucille (B.B.King; 0:00 – 0:41)<br>https://www.youtube.com/watch?v=-Y8QxOjuYHg	
23	Welches Instrument spielt hier im Vordergrund?	
	a) Trompete<br>b) Keyboard<br>c) Gitarre<br>d) Bass	
24	Aufnahme Michael Jackson „Thriller“ (0:49 – 1:26)<br>https://www.youtube.com/watch?v=ZEHsIcsjtdI	
24	Welchen Sänger hören Sie?	
	a) Prince<br>b) King<br>c) Michael Jackson<br>d) Elvis Presley	
25	Mozart Klavierquartett g-moll K. 478 Andante (0:00 – 1:05)<br>https://www.youtube.com/watch?v=oyUZizmKwCY	
25	Welches Instrument spielt hier **nicht** mit?	
	a) Klavier<br>b) Geige<br>c) Cello<br>d) Fagott	
26	Non, je ne regrette rien (Edith Piaf; 0:00 – 0:43)<br>https://www.youtube.com/watch?v=t6wjCcWC2aE	
26	Es handelt sich bei diesem Lied um folgende Gattung:	
	a) Michael Jackson-Stil<br>b) Tangogesang<br>c) Vaudeville-Komödie<br>d) Französisches Chanson	

27	Almost blue (Chet Baker; 0:00 – 1:11)<br>https://www.youtube.com/watch?v=z4PKzz81m5c	
27	Welches Instrument spielt hier die Solostimme?	
	a) Flöte<br>b) Oboe<br>c) Trompete<br>d) Violine	
28	Camille Saint-Saens: Fantasie in a-Moll op. 95; 0:00 – 0:25<br>https://www.youtube.com/watch?v=V7cxZSNvvKs	
28	Welches Instrument spielt hier?	
	a) Klavier<br>b) Gitarre<br>c) Cembalo	

	d) Harfe	
29	Rhapsody in Blue (Gershwin; 0:00 – 0:41)<br>https://www.youtube.com/watch?v=ynEOo28lsbc	
29	Welches ist das dominierende Instrument?	
	a) Mundharmonika<br>b) Horn<br>c) Tuba<br>d) Klarinette	
30	Winterreise von F. Schubert (Am Brunnen vor dem Tore; 0:00 – 0:48)<br>https://www.youtube.com/watch?v=zC7gEVSgf9k	
30	Dieses Lied gehört zur Gattung?	
	a) Kunstlied<br>b) Konzertarie<br>c) Volkslied<br>d) Folklorelied	
31	Reggae-Bob Marley – “I shot the sheriff” (0:00 – 0:40)<br>https://www.youtube.com/watch?v=2XiYUYcpsT4	
31	Zu welchem Musikstil gehört dieser Song?	
	a) Rap<br>b) Blues<br>c) Folk<br>d) Reggae	
32	Trio Flöte-Cello Klavier von Martinû; (0:00 – 0:51)<br>https://www.youtube.com/watch?v=G_9nVtlq64Y	
32	Wie viele Instrumente spielen mit?	

	a) 2<br>b) 3<br>c) 4<br>d) 5	
33	Mendelssohn : Violinkonzert e-moll Anfang 1. Satz; (0:00 – 0:35)<br>https://www.youtube.com/watch?v=t_uA_W1wB5A	
33	Dieses Stück ist …	
	a) eine Symphonie<br>b) ein Streichquartett<br>c) Ein Geigenkonzert<br>d) Ein Flötenkonzert	
34	Mozart, Zauberflöte: Arie der „Königin der Nacht“ (0:00 – 0:35)<br>https://www.youtube.com/watch?v=Zt56hgQe1co	
34	Welche menschliche Stimmlage hören Sie?	
	a) Tenor<br>b) Bass<br>c) Alt<br>d) Sopran	
35	Traditionelle chinesische Musik; 0:00 – 0:40	
	https://www.youtube.com/watch?v=E5LGXhcN7tg	
35	Was für eine Art von Musik hören Sie hier?	
	a) Traditionelle chinesische Musik<br>b) Afrikanische Musik<br>c) musique balinaise<br>d) musique du Congo	
36	Beethoven, Fünfte Symphonie (0:00 – 0:45)<br>https://www.youtube.com/watch?v=fOk8Tm815lE	
36	Der Komponist dieser Musik ist:	
	a) Ludwig v. Beethoven<br>b) Joseph Haydn<br>c) Peter Tchaikovsky<br>d) Wolfgang Amadeus Mozart	
37	Yesterday von The Beatles; 0:00 – 0:39<br>https://www.youtube.com/watch?v=WSuVCyT63II	
37	Von welcher Gruppe ist dieses Lied?	
	a) Rolling Stones<br>b) Beatles<br>c) Bee Gees<br>d) Michael Jackson	
38	Helene Fischer: Atemlos; 0:00 – 0:30<br>https://www.youtube.com/watch?v=Pv0AEc3w3uU	

38	Welche Sängerin hören Sie?	
	a) Edith Piaf<br>b) Mireille Matthieu<br>c) Amy Winehouse<br>d) Helene Fischer	
39	Badinerie von Bach (0:00 – 0:32)<br>https://www.youtube.com/watch?v=Tv40mcAM1ZA	
39	Welches Soloinstrument?	
	a) Flöte<br>b) Cembalo<br>c) Geige<br>d) Oboe	
40	Jellicle songs (Cats, 0:00 – 1:03)<br>https://www.youtube.com/watch?v=rYAeA8iREsM	
40	Dieses Lied ist Teil einer/s	
	a) Oper<br>b) Musicals<br>c) Operette<br>d) Liederkreises	
41	Benny Goodman (China Boy; 0:00 – 0:36)<br>https://www.youtube.com/watch?v=GuyzYAmMK-k	
41	Bei dieser Musik handelt es sich um	
	a) Soul<br>b) Reggae<br>c) Swing<br>d) Blues	
42	Bach – Wohltemperiertes Klavier, Fuge c-moll, 1. Band (0:00 – 0:44)<br>https://www.youtube.com/watch?v=hiCbnW5DUBo	
42	Bei dieser Klaviermusik handelt es sich um eine	
	a) Sonate<br>b) Fuge<br>c) Invention<br>d) Fantasie	
43	Bach: Musikalisches Opfer, 1. Ricercar à 3 (0:00 – 1:10)<br>https://www.youtube.com/watch?v=DdqGNCkKwgA	
43	Wie viele Themeneinsätze hören Sie?	
	a) Zwei<br>b) drei<br>c) vier<br>d) fünf	
44	Indischer Raga (0:00 – 0:35)<br>https://www.youtube.com/watch?v=DofUwZjMLeQ	

44	Diese Musik stammt aus	
	a) Zentralkongo<br>b) Bali<br>c) Indien<br>d) Peru	
45	Hound dog (Elvis Presley, 0:00 – 0:32)<br>https://www.youtube.com/watch?v=-eHJ12Vhpyc	
45	Hier hören Sie den „King of …“?	
	a) Pop<br>b) Rock‘n‘Roll<br>c) Swing<br>d) Jazz	
46	Schubert: Winterreise, Der Leiermann (0:32 – 1:32)<br>https://www.youtube.com/watch?v=ZTu_Jreo1SQ	
46	Wie wird die im Liedtext vorkommende Leier von der Klavierstimme musikalisch aufgegriffen?	
	a) durch Akkordbrechungen (Arpeggio)<br>b) durch schnelle Tonwiederholungen<br>c) durch ständige Wiederholung eines dreitönigen Motivs<br>d) durch wiederholte Liegetöne im Quintabstand	
47	Tschaikowski: Sinfonie Nr. 6, 2. Satz; 0:00 – 0:54<br>https://www.youtube.com/watch?v=GvEzdij0dlk	
47	In welcher Tatkart steht das gehörte Stück?	
	a) 2/4-Takt<br>b) 3/4-Takt	
	c) 5/4-Takt<br>d) 7/4-Takt	
48	Vivaldi: Sommer, 1. Satz (ab Einsatz der Solo-Violine; 1:14 – 1:40)<br>https://www.youtube.com/watch?v=QpsWrLa24Os	
48	Welches Tiergeräusch wird hier musikalisch nachgeahmt?	
	a) Der Ruf des Kuckucks<br>b) Das Wiehern der Pferde<br>c) Das Bellen der Hunde<br>d) Das Zirpen der Grille	
49	Sonne (Rammstein; 0:00 – 0:45)<br>https://www.youtube.com/watch?v=YtEWoavDlcM	
49	Welche deutsche Rockband ist hier zu hören:	
	a) Scorpions<br>b) Wolfgang Petry<br>c) Herbert Grönemeyer<br>d) Rammstein	

50	2. Sinfonie 1. Satz von Gustav Mahler (2:50 – 3:48)<br>https://www.youtube.com/watch?v=z2KcsjA_PEQ	
50	Welche Begrifflichkeiten treffen hier zu:	
	a) Musikdrama<br>b) Spätromantik<br>c) nationaler Musikstil<br>d) Atonalität	
51	Paul Desmond: „Take Five“, 0:00 – 0:50<br>https://www.youtube.com/watch?v=vmDDOFXSgAs	
51	Diese Melodie hat folgende Eigenschaften:	
	a) rockig<br>b) swingig<br>c) Soul<br>d) Bebop	
52	f-moll Klaviersonate von Beethoven op. 1, 0:00 – 0:35<br>https://www.youtube.com/watch?v=H-PuqndNGV4	
52	Dieses Klavierstück bezeichnet man als	
	a) Charakterstück<br>b) Klaviersonate<br>c) Nocturne<br>d) Etüde	
53	Ballade g-moll von Chopin (0:23 – 0:57)<br>https://www.youtube.com/watch?v=wgPh3mSYf0M	
53	Diese Stück hat komponiert:	
	a) Johannes Brahms<br>b) Wolfgang Amadeus Mozart<br>c) Frédéric Chopin<br>d) Arnold Schönberg	

54	Kaiser-Walzer von Johann Strauss (7:05 – 7:47)<br>https://www.youtube.com/watch?v=FkoRSojz7_g	
54	In welcher Taktart steht dieses Stück?	
	a) Dreier-Takt<br>b) Vierer-Takt<br>c) Zweier-Takt<br>d) Fünfer-Takt	
55	Musik mit Theremin; 0:00 – 0:42<br>https://www.youtube.com/watch?v=PjnaciNT-wQ	
55	Das dominierende Musikinstrument nennt man	
	a) Synthesizer<br>b) Elektronischer Röhrenverstärker<br>c) Sphärophon<br>d) Theremin	

56	Brahms 1. Klavierkonzert d-moll Anfang; 0:00 – 0:57<br>https://www.youtube.com/watch?v=Fu4-Enucukk	
56	Diese Musik gehört stilistisch zur	
	a) Romantik<br>b) Klassik<br>c) Barock<br>d) Klassische Moderne	
57	Aufnahme der Geissenklösterle-Flöte (0:00 – 0:46)<br>http://www.edge-cdn.net/video_1236592?playerskin=37016	
57	Das Erstaunliche an der Musik auf dieser rekonstruierten steinzeitlichen Flöte ist:	
	a) Lautstärke<br>b) Tonskala enthält Halb- und Ganztonabstände<br>c) nur drei unterschiedliche Töne können produziert werden<br>d) Virtuosität	
58	Maurice Ravel – Daphnis et Chloé (1:39 – 2:40)<br>https://www.youtube.com/watch?v=YHrstmOPKBQ	
58	Zu welcher Stilrichtung gehört diese Musik?	
	a) Impressionismus<br>b) Musik des Barock<br>c) Musik der Wiener Klassik<br>d) Renaissance	
59	David Garrett, Crossover Stück Vivaldi (0:00 – 0:52)<br>https://www.youtube.com/watch?v=y2YibV3xgM8	
59	Man nennt diesen Stil	
	a) Crossover<br>b) Garretism<br>c) Barock<br>d) Neoklassizismus	
60	Enrico Morricone, Mundharmonika (Spiel mir das Lied vom Tod; 0:00 – 0:37)<br>https://www.youtube.com/watch?v=zIlT0wkW9Vg	

60	Das besondere an dieser Filmmusik ist	
	a) Virtuoser Einsatz der Mundharmonika<br>b) Leitmotiv-Technik<br>c) Zahlreiche Zitate aus Wagner Opern<br>d) Virtuoser Einsatz der Celli	
