# Supplementary material for: Acquisition of musical skills and abilities in older adults—results of 12 months of music training
Source: BMC Geriatr. 2024 Dec 19;24:1018. doi: 10.1186/s12877-024-05600-2 (PMC11658158; doi:10.1186/s12877-024-05600-2)
Supplement: Supplementary file 3 — Supplementary Material 3. [file 12877_2024_5600_MOESM3_ESM.pdf]

Ode to Joy simple

« Hymne à la joie » / *An die Freude*

Simple

Symphonie no. 9 - 4e mouvement

(v3) - op. 125

♪ = 112

Ludwig van Beethoven (1770 - 1827)
arr. Xavier Favier

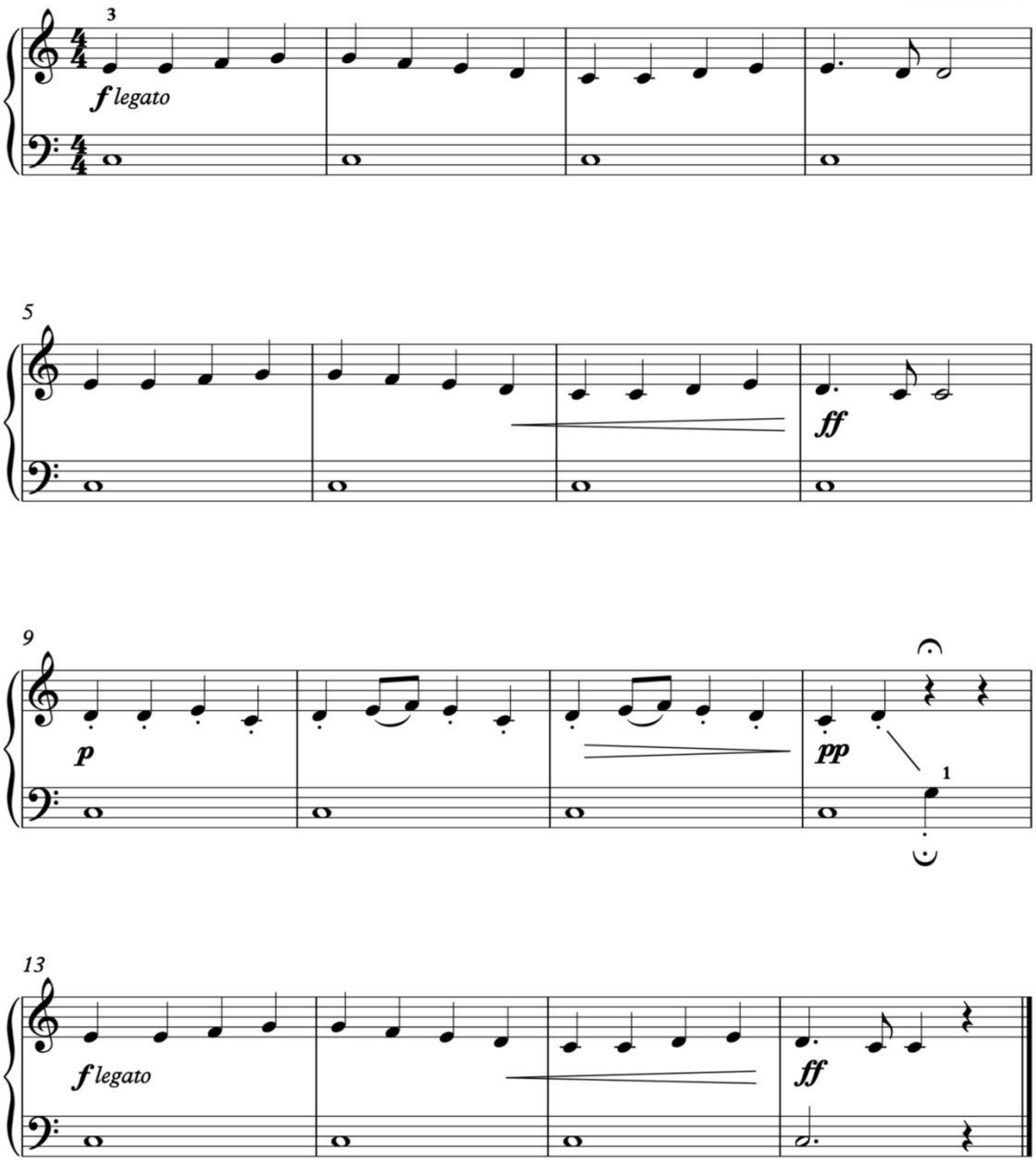

morceau HEdS mars 2019

Ode to Joy advanced

« Hymne à la joie » / *An die Freude*

Normal

Symphonie no. 9 - 4e mouvement

(version normale) - op. 125

Allegro assai

Ludwig van Beethoven (1770 - 1827)

arr. Xavier Favier

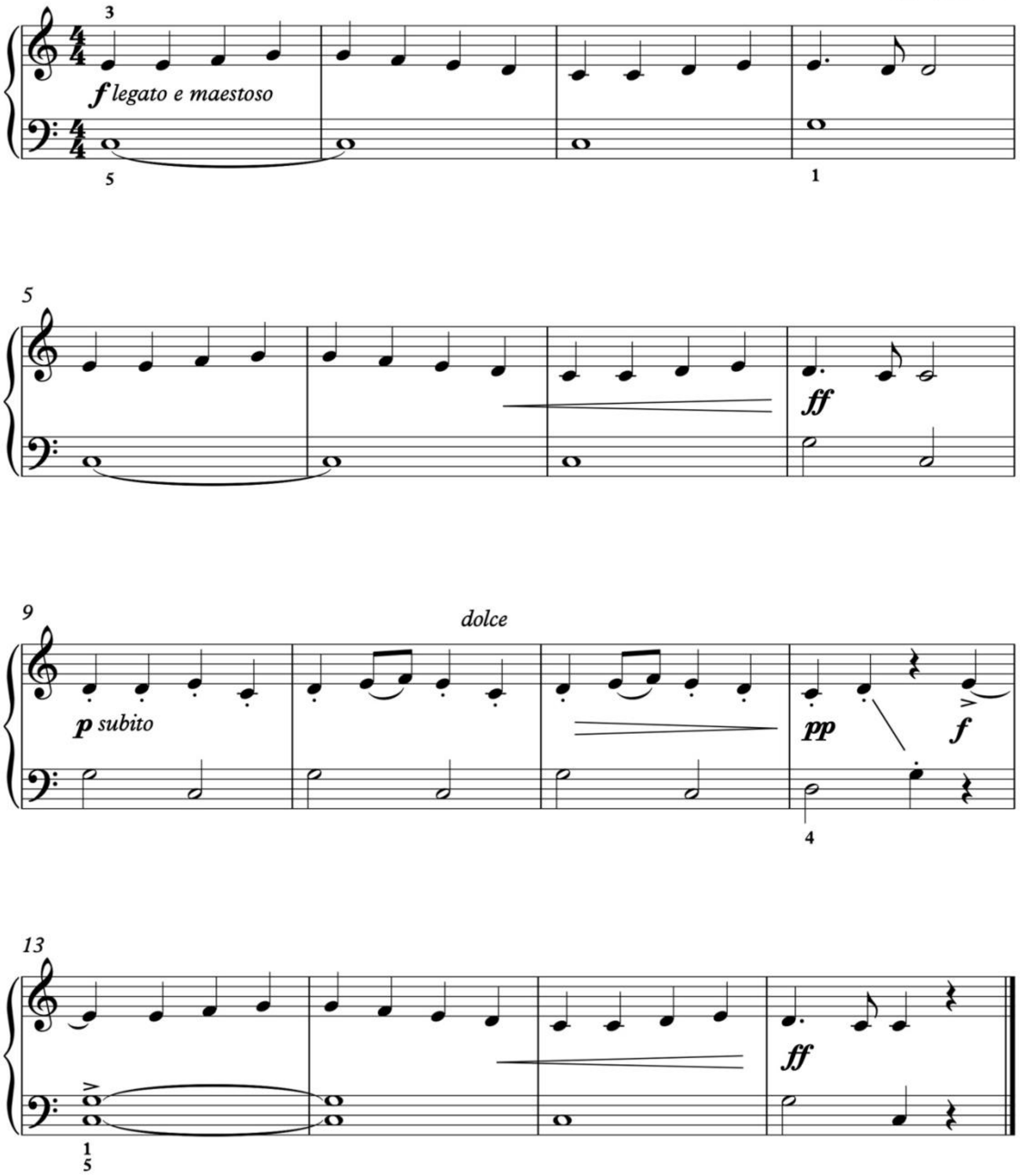

morceau HEdS mars 2019
