# Supplementary material for: Acquisition of musical skills and abilities in older adults—results of 12 months of music training
Source: BMC Geriatr. 2024 Dec 19;24:1018. doi: 10.1186/s12877-024-05600-2 (PMC11658158; doi:10.1186/s12877-024-05600-2)
Supplement: Supplementary file 4 — Supplementary Material 4. [file 12877_2024_5600_MOESM4_ESM.pdf]

Evaluation Scale for "Ode to Joy"

First Time Listening

Articulation: The performance was accurate with respect to articulation, e.g., staccato and legato were played when indicated in the score.

Rhythm: The performance was accurate with respect to rhythm.

Dynamics: The performance was accurate with respect to dynamics, i.e., participants differentiated between forte and piano etc.

Second Time Listening

Accuracy of Notes (Pitch): The performance was accurate with respect to pitch, i.e., the correct notes were played.

Fluency: The performance was played without interruptions.

Expressivity: The performance communicated an understanding of expressive issues.

Agreement Likert Scale (1-7):

1	2	3	4	5	6	7
Strongly Disagree	Disagree	Somewhat Disagree	Neutral	Somewhat Agree	Agree	Strongly Agree
