# Supplementary material for: Acquisition of musical skills and abilities in older adults—results of 12 months of music training
Source: BMC Geriatr. 2024 Dec 19;24:1018. doi: 10.1186/s12877-024-05600-2 (PMC11658158; doi:10.1186/s12877-024-05600-2)
Supplement: Supplementary file 5 — Supplementary Material 5. [file 12877_2024_5600_MOESM5_ESM.pdf]

Rater Consistency Analysis: Intra- and Interrater Correlations

With the double rated evaluations, the Intra-correlation coefficients (IntraCC) were computed to determine the level of consistency among the raters' evaluations. The Table 1 presents the IntraCCs for each rater across the six variables.

Table 1: IntraCC of Each Rater for Each Variable

Rater	Fluency	Pitch	Rhythm	Articulation	Expressivity	Dynamics	**mean**
1	0.93	0.73	0.82	0.82	0.53	0.88	0.79
2	0.91	0.74	0.83	0.87	0.45	0.83	0.77
3	0.89	0.81	0.59	0.64	0.71	0.49	0.69
4			0.63	0.72		0.38	0.58
5	0.92	0.82	0.68	0.72	0.40	0.65	0.70
6	0.86	0.91	0.82	0.85	0.80	0.47	0.79
7	0.82	0.80	0.77	0.80	0.76	0.73	0.78
8	0.80	0.78	0.94	0.86	0.80	0.77	0.83
9	0.81	0.88	0.77	0.80	0.91	0.92	0.85
**mean**	0.87	0.81	0.76	0.79	0.67	0.68	

> .9; excellent, .75 - .9; good, .5 - .75; moderate, < .5; poor reliability (Koo & Li, 2016)

The IntraCCs indicated varying degrees of consistency among the raters for each variable. For fluency, the IntraCCs ranged from 0.80 to 0.93, indicating a high level of consistency among the raters' assessments. Similarly, pitch exhibited strong consistency among the raters, with IntraCCs ranging from 0.73 to 0.91. Rhythm evaluations also demonstrated a high level of consistency, with IntraCCs ranging from 0.59 to 0.94. Articulation was assessed with moderate to high reliability, as indicated by IntraCC values ranging from 0.64 to 0.86. Expressivity showed moderate consistency among the raters, with IntraCCs ranging from 0.40 to 0.91. The evaluations of dynamics exhibited the greatest variability among the raters, with IntraCCs ranging from 0.38 to 0.92. Overall, the results suggest that the raters

demonstrated good to strong consistency in evaluating fluency, pitch, rhythm, and articulation. However, there was more variability in the assessments of expressivity and dynamics.

The IntraCCs reveal considerable variability in consistency among the raters. For instance, Rater 8 and Rater 9 demonstrate higher reliability compared to Rater 3 and Rater 4. Despite analyzing factors such as age, years of piano practice or teaching, no clear explanations for these differences were found, likely due to the small group size and alignment of raters in these factors. Furthermore, no differences were observed between different university degrees, such as music educators and musicians, in terms of rating. In the subsequent analysis, the ratings were weighted based on the IntraCCs per rater and variable. This approach ensured that raters with higher consistency had a greater impact on the analysis of the aspects of piano performance compared to raters with lower IntraCCs.

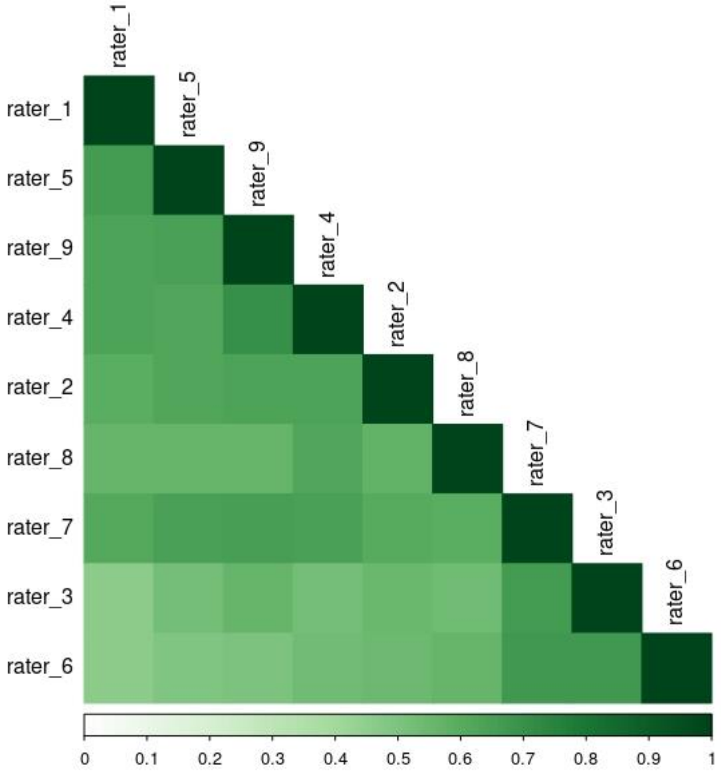

Figure 1: Interrater Correlation

The Interrater Correlation Coefficients (InterCC) also showed moderate to high reliability among all raters (see

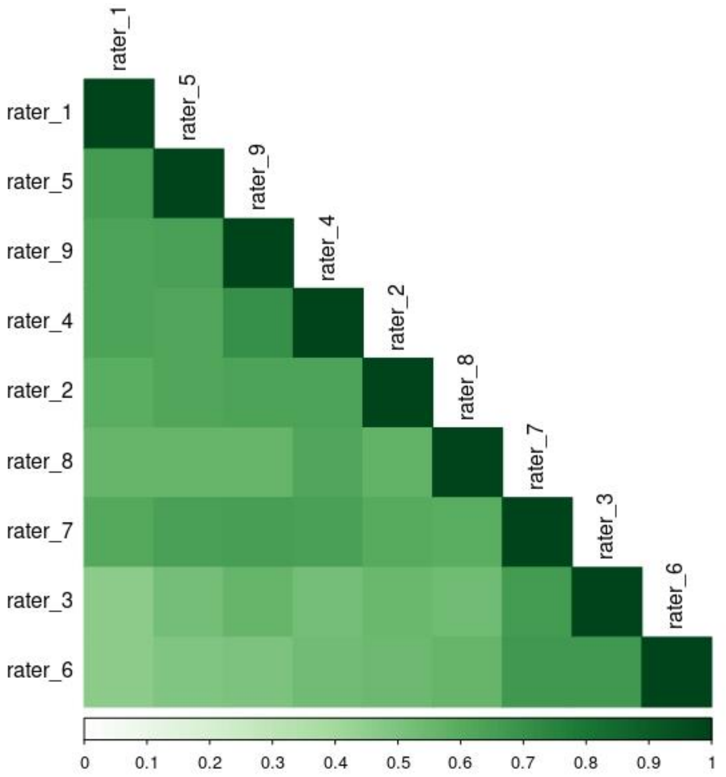

Figure 1). Among the raters, no outlier could be found. As shown in Table 2 the correlation coefficients are between 0.78 and 0.93. Expressivity shows the lowest reliability of 0.78, still being in a good range. The raters were the most consistent on fluency, as shown by the correlation score of 0.93. Articulation and Pitch follow directly.

Table 2: InterCC for each Variable

	Articulation	Dynamics	Rhythm	Pitch	Fluency	Expressivity
InterCC	0.92	0.87	0.87	0.90	0.93	0.78

> .9; excellent, .75 - .9; good, .5 - .75; moderate, < .5; poor reliability (Koo & Li, 2016)
