# Supplementary material for: Acquisition of musical skills and abilities in older adults—results of 12 months of music training
Source: BMC Geriatr. 2024 Dec 19;24:1018. doi: 10.1186/s12877-024-05600-2 (PMC11658158; doi:10.1186/s12877-024-05600-2)
Supplement: Supplementary file 6 — Supplementary Material 6. [file 12877_2024_5600_MOESM6_ESM.pdf]

Factor Analysis of Piano Ratings: Latent Variable Relationships

Scree plot analysis (see figure) demonstrated the clear existence of one single latent factor, referred to as *piano performance*. The items of which piano performance consist could be extracted through the model fit of CFA models as shown in Table 1. When extracting expressivity and dynamics from the model, the fit clearly improved to a very good fit of 0.98 in CFI and TLI and 0.1 in RSMEA. Further, the MILCS model (see Figure 1) was generated by only four variables: articulation, rhythm, pitch, and fluency.

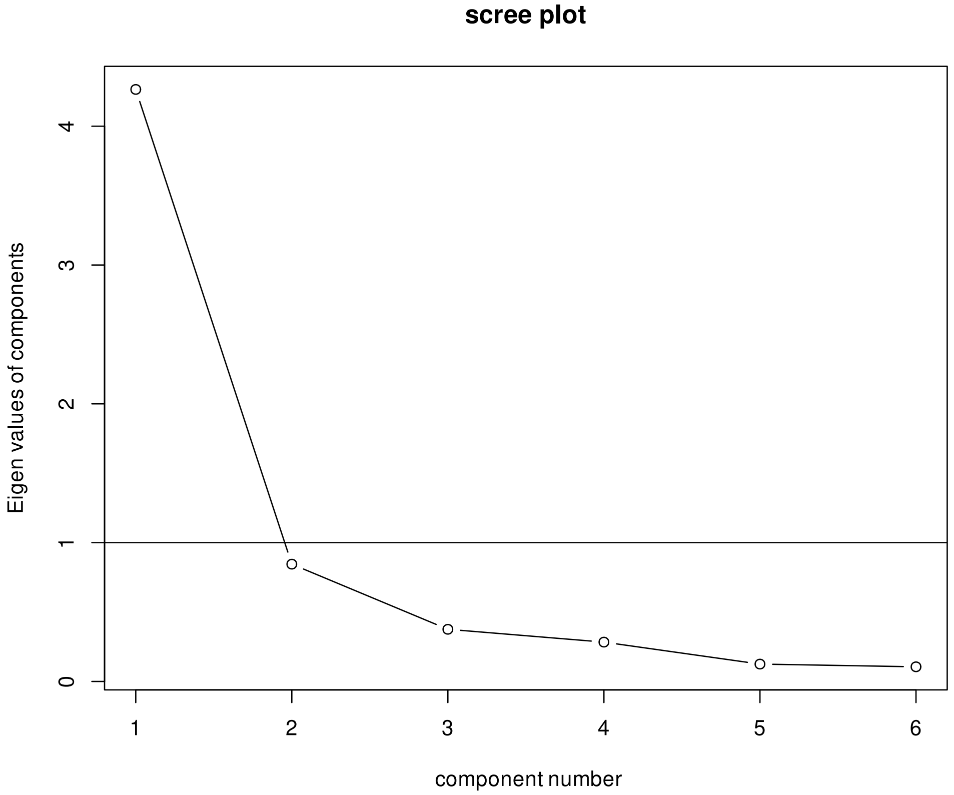

Table 1: Fit of CFA Models

Piano performance =	Chi-Square	CFI	TLI	RMSEA
A + R + D + P + F + E	0.00	0.92	0.89	0.18
A + R + P + F	0.01	0.98	0.97	0.11

The model's overall fit statistics indicate a favorable fit for the MILCS model. The CFI and TLI values of approximately 0.98 suggest a very good fit, as do the RMSEA values of around 0.1. For both measurement time points, scree plots consistently supported a single latent variable

underlying all piano variables. The standardized loadings represent the strength of the relationships between latent variables and observed indicators. At three months, articulation, rhythm, pitch, and fluency had relatively strong loadings, ranging from 0.55 to 0.91. These loadings further strengthened slightly at 12 months, ranging from 0.57 to 0.97. The variables' loadings only marginally changed, with slight improvements of 0.02 to 0.04. Collectively, these indicators effectively signify both latent variables, *piano performance* at both three and 12 months. The regression weight between the *piano performance* at three and 12 months reveals that the former predicts the latter quite effectively. The coefficient of 1.10 indicates that a unit increase in *piano performance* at 12 months is associated with a 1.10 unit increase in *piano performance* at 3 months.

The positive covariance between the variables at both time points indicate temporal correlations among the indicators. Covariance values range from 0.03 for rhythm to 0.34 for articulation. For pitch, the covariance is 0.18, and for fluency 0.14. For each variable, this means that higher scores at three months align with higher scores at 12 months. The covariance between *piano performance* at three months and the change score (representing the latent variable between *piano performance* at 3 and 12 months) is negative, at -0.03. This implies that as the performance at three months increases, the magnitude between the performance of three and 12 months tends to decrease. Regarding variances, the values for each latent variable elucidate the proportion of variability explained in the indicators. The variance of 0.01 for the change of *piano performance* over the year suggests its explanatory power of approximately 1% over its indicators.

The R-Square values shed light on the extent to which variability in latent variables is accounted for by the indicators. For *piano performance* at 12 months, the indicators explain 100% of the variability. For *piano performance* at three months, articulation contributes around 30%, while pitch contributes around 60%, and rhythm and fluency each contribute approximately 85% to the variance. This pattern remains consistent in *piano performance* at 12 months, with articulation explaining 33%, pitch explaining 69%, fluency 87% and rhythm explaining 94% of variance.

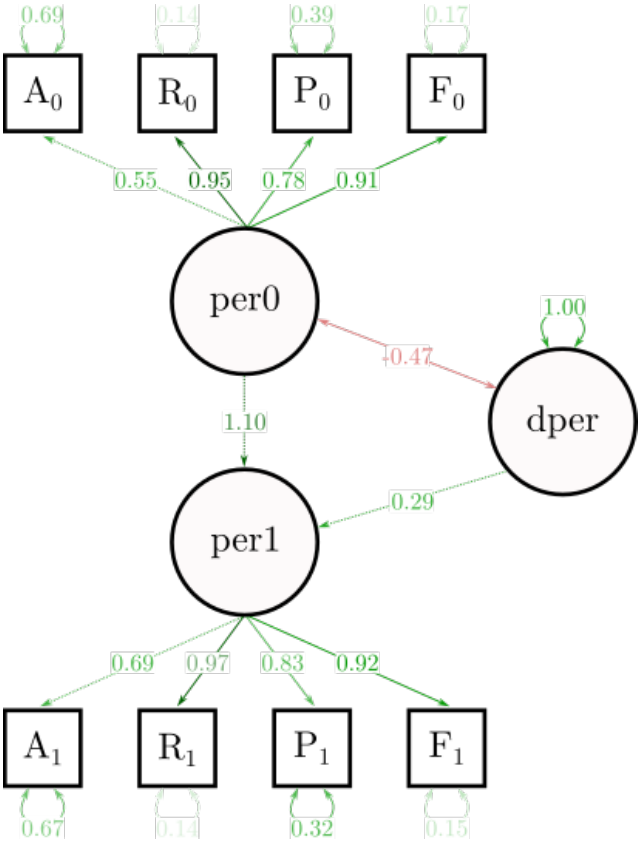

Figure 1: Multiple Indicator Univariate Latent Change Score. Multiple Indicator Univariate Latent Change Score The path model shows the loadings of the observed variables (A: articulation, R: rhythm, P: pitch, F: fluency) on the latent variables of piano performance at three months (per0) and at 12 months (per1). For each variable, the residual variance and covariance are displayed. Additionally, die change score is shown (dper). Further explanation in the text.
